# Recombinant HSA-CMG2 Is a Promising Anthrax Toxin Inhibitor

**DOI:** 10.3390/toxins8010028

**Published:** 2016-01-20

**Authors:** Liangliang Li, Qiang Guo, Ju Liu, Jun Zhang, Ying Yin, Dayong Dong, Ling Fu, Junjie Xu, Wei Chen

**Affiliations:** 1 Laboratory of Vaccine and Antibody Engineering, Beijing Institute of Biotechnology, Beijing 100071, China; byebyelll@163.com (L.L.); jackyguo@163.com (Q.G.); 18701522421@163.com (J.L.); justforhere@126.com (J.Z.); yinying1028@sina.cn (Y.Y.); akta100@sina.com (D.D.); fuling3436@163.com (L.F.); 2 Center for Disease Control and Prevention of Navy, Beijing 101113, China

**Keywords:** *Bacillus anthracis*, protective antigen, anthrax toxin inhibitor, CMG2, HSA

## Abstract

Anthrax toxin is the major virulence factor produced by *Bacillus anthracis*. Protective antigen (PA) is the key component of the toxin and has been confirmed as the main target for the development of toxin inhibitors. The inhibition of the binding of PA to its receptor, capillary morphogenesis protein-2 (CMG2), can effectively block anthrax intoxication. The recombinant, soluble von Willebrand factor type A (vWA) domain of CMG2 (sCMG2) has demonstrated potency against anthrax toxin. However, the short half-life of sCMG2 *in vivo* is a disadvantage for its development as a new anthrax drug. In the present study, we report that HSA-CMG2, a protein combining human serum albumin (HSA) and sCMG2, produced in the *Pichia pastoris* expression system prolonged the half-life of sCMG2 while maintaining PA binding ability. The IC_50_ of HSA-CMG2 is similar to those of sCMG2 and CMG2-Fc in *in vitro* toxin neutralization assays, and HSA-CMG2 completely protects rats from lethal doses of anthrax toxin challenge; these same challenge doses exceed sCMG2 at a sub-equivalent dose ratio and overwhelm CMG2-Fc. Our results suggest that HSA-CMG2 is a promising inhibitor of anthrax toxin and may contribute to the development of novel anthrax drugs.

## 1. Introduction

*Bacillus anthracis*, a zoonotic pathogen, is considered as a potential biological agent and biologic terrorism pathogen due to the strong environmental persistence of its spores [1]. Anthrax toxin, the main virulence factor produced by *Bacillus anthracis*, is composed of three proteins: protective antigen (PA), lethal factor (LF), and edema factor (EF) [2]. LF is a Zn^2+^-dependent metallo-enzyme that can inhibit the activation of the MAPKK signaling pathway [3]. EF is a Ca^2+^-dependent adenylate cyclase that can up-regulate the cytoplasmic cAMP level [4]. PA is able to mediate the cell entry of LF and EF by binding to cell membrane receptors [5]. After cleavage by protease at the cell membrane or in the plasma, the 63-kDa fragment of PA forms a heptamer, exposing LF/EF binding sites [6]. After the binding of up to three molecules of LF/EF, the entire complex is then internalized via receptor-mediated endocytosis. Acidification in the endosome promotes the transformation of the pre-pore complex into the pore complex and the translocation of the catalytic LF and/or EF molecules into the cell cytosol. Individually, PA, LF, and EF are non-toxic; however, they can form the toxic PA/LF (LT, lethal toxin) and PA/EF (ET, edema toxin) complexes on the cell surface, which can then be transported to the cytoplasm.

There are two anthrax toxin receptors, tumor endothelial marker 8/anthrax toxin receptor 1 (TEM8/ANTXR1) [7] and capillary morphogenesis protein 2/anthrax toxin receptor 2 (CMG2/ANTXR2) [8]. The exact physiological functions of these two proteins are not well known. Each receptor contains a signal peptide, a single extracellular von Willebrand factor A (vWA) domain where PA binds directly, a single-pass transmembrane region (TM) for plasma membrane anchoring, and a cytosolic tail. Soluble fragments of receptors, such as the vWA domain of CMG2 (sCMG2), have been reported to inhibit PA-receptor binding [9,10]. Initially identified in human placenta tissue, CMG2 specifically binds human collagen IV and lamina. The expression of CMG2 is specifically up-regulated in human umbilical vein endothelial cells and was shown to be highly induced during collagen capillary formation [11]. Although the vWA domains of CMG2 and TEM8 share 60% of the same residues, recent studies indicated that CMG2 plays a major role in death caused by anthrax toxin, whereas the role of TEM8 is minor [12]. Furthermore, the IC_50_ of soluble CMG2 determined by a cell toxin inhibition assay was 11.4-fold lower than that of TEM8 (expressed from human FreeStyle 293 cell), while the affinity of soluble CMG2 for PA measured by SPR (Surface Plasmon Resonance) was 1000 fold stronger than that of TEM8 (expressed from human FreeStyle 293 cell) [9]. Therefore, we chose to study CMG2 as an anthrax toxin-specific therapeutic drug.

Anthrax toxin is primarily treated with antibiotics. Anthrax infection, especially in its inhalational form, is difficult to treat because the appearance of symptoms indicates that a large quantity of anthrax toxin is present in the human body [13]. Even though antibiotic therapy can inhibit bacterial growth, the infection can still be lethal due to the accumulation of toxins [14]. Therefore, an effective post-exposure approach to treating anthrax would include both antibiotics and anthrax toxin inhibitors [15].

Anthrax toxin inhibitors including monoclonal antibodies, receptor decoys, AIGIV (anthrax intravenous immunoglobulin), and small-molecule inhibitors are a hot area of study in the field of biological safety [16]. Compared with monoclonal antibodies, which may not bind epitope-mutated PA, the receptor decoy sCMG2 binds both wild-type and mutant forms of PA [17]. However, the half-life of sCMG2 in HSD rats is only about 10 min, which is too short for it to be employed as a protein drug [18]. One method to prolong the half-life of sCMG2 is the combination of sCMG2 with the Fc fragment of human immunoglobulin (CMG2-Fc). In one study, the fused protein protected mice infected with *Bacillus anthracis* spores from death, even when the mice were re-injected with spores 30 days after the administration of CMG2-Fc [19]. In contrast, another study suggested that CMG2-Fc only prolonged the half-life of CMG2, extending the survival time, but did not provide complete protection from death [18].

In this study, we constructed a new form of the fusion protein HSA-CMG2 combining human serum albumin (HSA) and sCMG2, measured its half-life and affinity for anthrax toxin, and evaluated its protection efficiency *in vitro* and *in vivo*. By comparing the results with the reported data for sCMG2 and CMG2-Fc, we suggested HSA-CMG2 as a new anthrax drug candidate.

## 2. Results

### 2.1. Expression and Purification of HSA-CMG2

**Figure 1:**
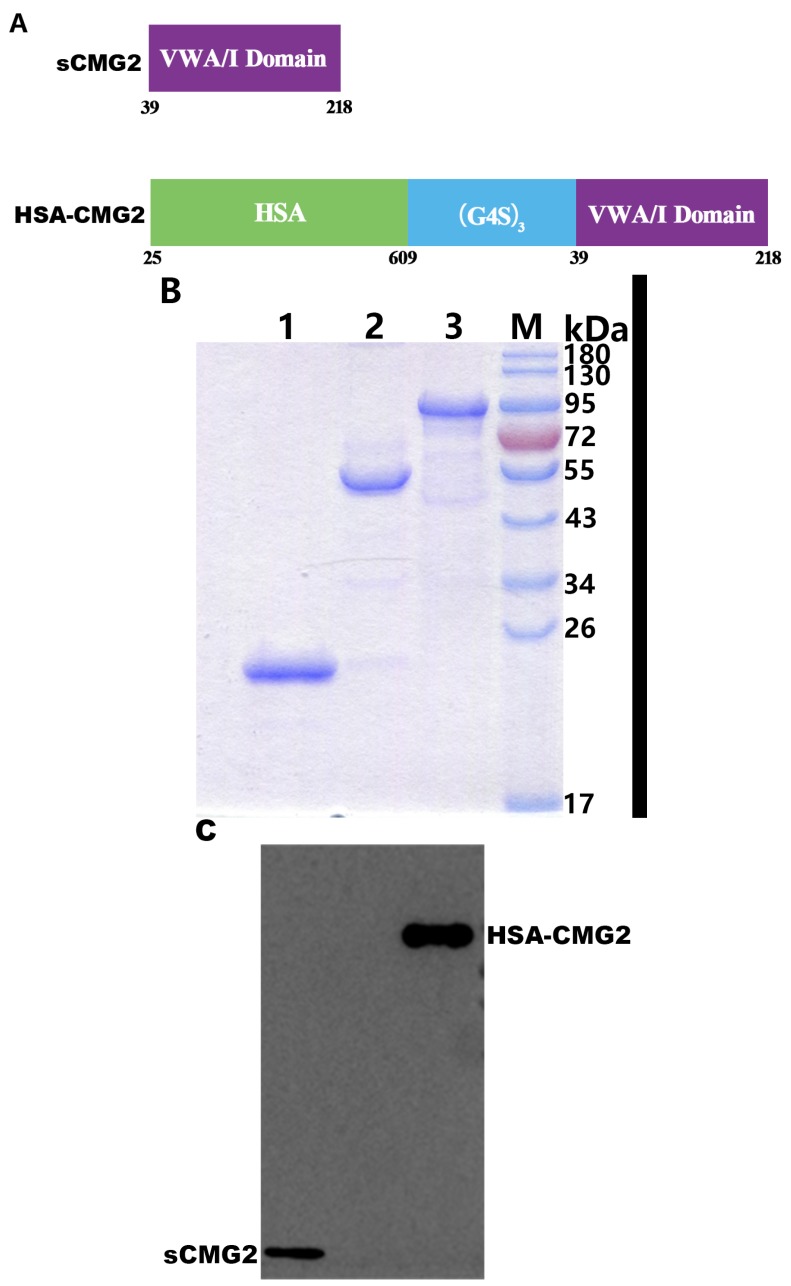
(**A**) Schematic showing the makeups of sCMG2 (aa 39–218) and HSA-CMG2 (fusion aa 25–609 of HSA and aa 39–218 of sCMG2). VWA/I domain: von Willebrand factor A/integrin-like I domain of CMG2; (G4S)_3_: linker GGGGS×3; (**B**) Purification and identification of sCMG2, CMG2-Fc and HSA-CMG2: Coomassie Brilliant Blue stained gels of sCMG2 (lane 1), CMG2-Fc (lane 2) and HSA-CMG2 (lane 3) on SDS-PAGE gels containing 12% polyacrylamide gel under reduced conditions. Molecular weight markers are indicated; (**C**) Western blotting of HSA-CMG2 (100 ng/lane) and sCMG2 (50 ng/lane) with mouse anti-CMG2 antibody.

In order to prolong the half-life of sCMG2, we constructed the fused protein HSA-CMG2 (Figure 1A). Due to its ability to highly express recombinant proteins and the simplicity of the associated operating procedures, *pichia yeast* was used to express HSA-CMG2, and the obtained expression level was approximately 200 mg/L. After the elimination of pigment using Blue Sepharose (GE) affinity purification and ion exchange chromatography on a Q-HP column (GE), the purity of HSA-CMG2 was approximately 95% (Figure 1B). The molecular weight was determined to be about 89-kDa, which is consistent with the theoretical value. Subsequently, sCMG2 from *E. coli* and CMG2-Fc from mammalian cells were purified and analyzed by SDS-PAGE followed by staining with Coomassie Brilliant Blue (Figure 1B). Western blotting demonstrated that the 89-kDa band was identified as anti-CMG2 antibody (Figure 1C).

### 2.2. Affinity of HSA-CMG2 for rPA

To determine whether the fused HSA influences the affinity CMG2 for PA, SPR assay was performed. The determined affinities of HSA-CMG2 and sCMG2 for rPA were 5.76 nmol/L and 1.67 nmol/L, respectively. Although the affinity of HSA-CMG2 for rPA was weaker than that of sCMG2 for rPA (Table 1), both affinities were on the nM level; thus, we inferred that the fusion of HSA had only a minor influence on the biological activity of CMG2.

**Table 1:** Kinetic data for the binding of rPA with HSA-CMG2.

Receptor Decoys	*k*_a_ (1/Ms × 10^6^)	*k*_d_ (1/s × 10^2^)	*K*_D_ (nmol/L)
sCMG2	7.98 ± 5.80	1.38 ± 1.02	1.67 ± 0.26
HSA-CMG2	0.26 ± 0.21	0.12 ± 0.06	5.76 ± 2.22

### 2.3. Half-Life of HSA-CMG2 and CMG2-Fc

**Figure 2:**
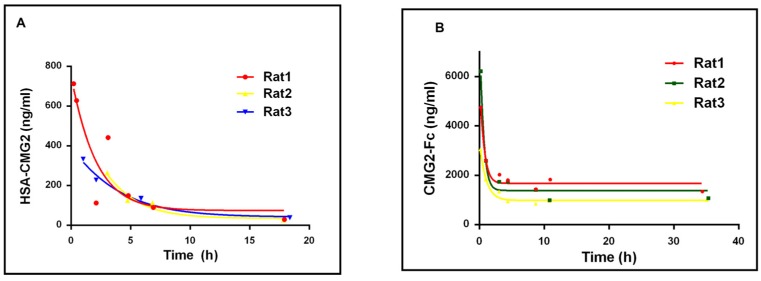
The concentrations of HSA-CMG2 (**A**) and CMG2-Fc (**B**) in rats *vs.* time after injection. Curve-fitting and half-life (h) was calculated using Prism (GraphPad, Inc., La Jolla, CA, USA).

The half-life of HSA-CMG2 in male SD rats was compared with that of CMG2-Fc measured by testing the concentrations of different samples at different times after injection with these proteins. The half-life of HSA-CMG2 measured as 5.01 ± 0.88 h (Figure 2A). In contrast, CMG2-Fc showed a much longer half-life of 29.98 ± 7.88 h (Figure 2B).

### 2.4. In Vitro Toxin Inhibition Activity of HSA-CMG2

**Figure 3:**
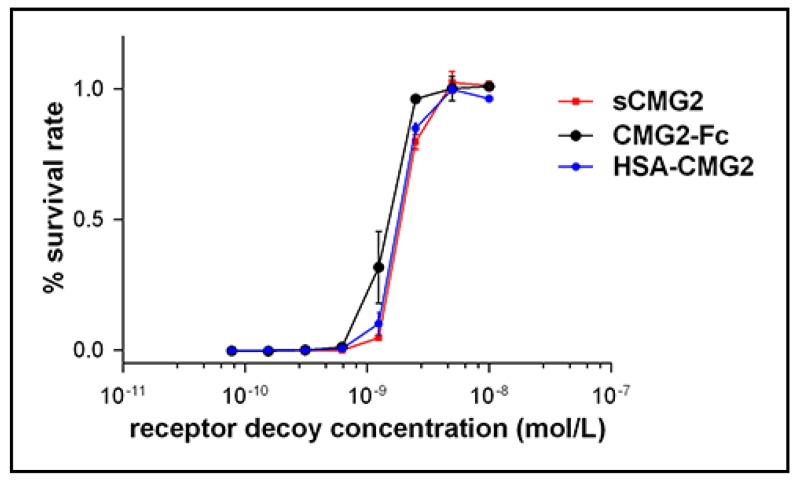
Inhibition of anthrax toxin activity *in vitro*. The experiments were performed at different receptor decoy concentrations due to the different abilities of the different receptor decoys to neutralize lethal toxin (LT). Each assay was performed three times with duplicates within each assay. The data points represent the mean ± SD values of the triplicate samples.

To evaluate the toxin inhibition activity of HSA-CMG2, *in vitro* LT neutralization assay was employed to measure the IC_50_ of HSA-CMG2 for J774A.1 cells [20]. J774A.1 cells were treated with mixtures containing fixed concentrations of LT (50 ng/mL rPA + 40 ng/mL rLF) in the presence of various concentrations of HSA-CMG2. As the control, sCMG2 or CMG2-Fc was mixed with LT (50 ng/mL rPA + 40 ng/mL rLF) and added to J774A.1 cells. Cell viability was measured at 570 nm/630 nm. The IC_50_ of HSA-CMG2 was 1.83 ± 0.18 nmol/L, whereas those of sCMG2 and CMG2-Fc were 2.03 ± 0.12 nmol/L and 1.45 ± 0.26 nmol/L, respectively. The ability of HSA-CMG2 to protect J774A.1 cells against LT challenge was equal to those of sCMG2 (*p* = 0.25) and CMG2-Fc (*p* = 0.10), indicating that HSA-CMG2 exhibits high biological activity (Figure 3).

### 2.5. Toxin Neutralization of HSA-CMG2 in Vivo

**Figure 4:**
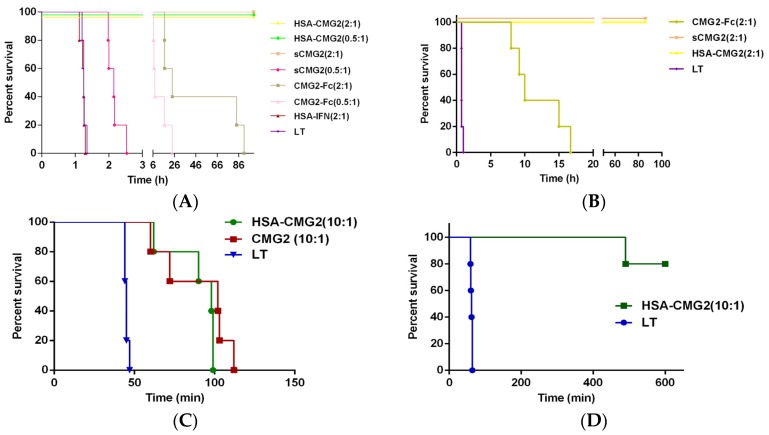

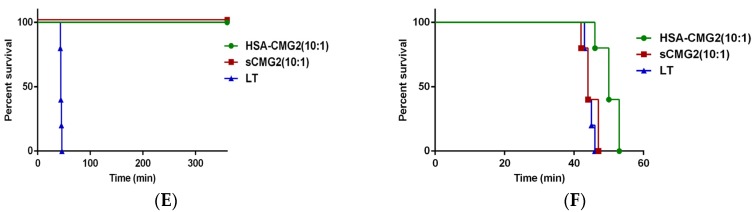
Receptor decoys protect rats from LT intoxication. (**A**,**B**) Male F344 rats (200–220 g, five per group) were co-injected intravenously with (**A**) LT **(**10 μg rPA + 5 μg rLF) and the receptor decoys and (**B**) LT (20 μg rPA + 10 μg rLF) and the receptor decoys. (**C**,**D**) For each rat, the receptor decoy was injected 5 min after (**C**) LT (20 μg rPA + 10 μg rLF) or (**D**) LT (10 μg rPA + 10 μg rLF) administration; (**E**,**F**) For each rat, the receptor decoy was injected, and LT (20 μg rPA + 10 μg rLF) was then injected 5 min (**E**) or 24 h (**F**) later via the tail vein. Ratios indicate the receptor decoy:rPA molar ratio.

To determine whether HSA-CMG2 can provide good *in vivo* protection for animals in addition to *in vitro* protection, animals were treated with a fixed concentration of LT (10 μg rPA + 5 μg rLF in 0.3 mL/200 g rat PBS, pH = 7.4). For rats that received receptor decoys, receptor decoys were also added to LT to a final volume of 0.3 mL. After incubating at room temperature for 10 min, the mixed solution was co-injected into the rats (see Table 2). The survival times of the HSA-CMG2 (receptor decoy:rPA molar ratios = 2:1 and 0.5:1) and sCMG2 (the receptor decoy:rPA molar ratio = 2:1) groups were significantly (*p* < 0.01) greater than that of the LT-only group. All rats in the HSA-CMG2 (the receptor decoy:rPA molar ratio, 2:1 and 0.5:1) and sCMG2 (the receptor decoy:rPA molar ratio, 2:1) groups remained alive until the final observation time, while all others were dead. CMG2-Fc did not protect the animals from death (Figure 4A); the survival times of the CMG2-Fc groups (receptor decoy:rPA molar ratios = 2:1 and 0.5:1) were 46.50 h and 12.37 h, respectively. An additional assay again indicated that HSA-CMG2 completely protected the animals, even at an increased dose of injected LT (20 μg rPA + 10 μg rLF; Figure 4B). Moreover, HSA-CMG2 (receptor decoy:rPA molar ratio = 10:1) and sCMG2 (receptor decoy:rPA molar ratio = 10:1) were injected 5 min after LT (20 μg rPA + 10 μg rLF per rat) administration. The survival times of the HSA-CMG2 and sCMG2 groups were significantly (*p* < 0.01) longer than that of the LT-only group. No significant difference (*p* > 0.05) was observed between the survival times of the HSA-CMG2 and sCMG2 groups (Figure 4C). The F344 rats were injected with LT (10 μg rPA + 10 μg rLF per rat) 5 min before HSA-CMG2 (receptor decoy:rPA molar ratio = 10:1) administration. At the end point of the experiment, one rat had died in the HSA-CMG2 group (Figure 4D). To determine whether the extended half-life of the HSA-CMG2 group resulted in prolonged protection, the rats were given receptor decoys at 5 min and 24 h before LT injection (20 μg rPA + 10 μg rLF per rat). Both HSA-CMG2 (receptor decoy:rPA molar ratio = 10:1) and sCMG2 (receptor decoy:rPA molar ratio = 10:1) protected all rats from death when LT was injected 5 min after receptor decoy administration (Figure 4E). When LT was injected 24 h after receptor decoy administration, even though all rats died, the survival time of the HSA-CMG2 group was significantly increased compared to those of the LT-only (*p* = 0.0084) and sCMG2 (*p* = 0.0069) groups. The survival time of the sCMG2 group (*p* = 0.5988) was similar to that of the LT group (Figure 4F). These results clearly indicate that HSA-CMG2 exhibits excellent *in vivo* biological activity and effectively protects rats against LT.

**Table 2:** *In vivo* protection against intoxication provided by different receptor decoys.

Inhibitor	Receptor Decoy:PA Moral Ratio	Survivors/Total	Average Time to Death (h)	Comparison of TTD (Unpaired *t*-Test) ^a^	Comparison of Survival Curve (Log-Rank Mantel-Cox) ^b^
HSA-CMG2	0.5:1	5/5	NA	-	*p* = 0.0018
HSA-CMG2	2:1	5/5	NA	-	*p* = 0.0018
sCMG2	0.5:1	0/5	2.17	*p* < 0.0001	*p* = 0.0018
sCMG2	2:1	5/5	NA	-	*p* = 0.0018
CMG2-Fc	0.5:1	0/5	12.37	*p* = 0.0122	*p* = 0.0018
CMG2-Fc	2:1	0/5	46.50	*p* = 0.0276	*p* = 0.0018
HSA-IFN	2:1	0/5	1.24	*p* = 0.5973	*p* = 0.7167
LT ^c^	-	0/5	1.34	-	-

NA, not applicable; TTD, time to death. ^a^ For comparison of the TTD with the LT-only control group by unpaired student’s *t-*test; ^b^ For comparison with the LT-only control group by log-rank (Mantel-Cox) test; ^c^ LT-only control group (10 µg of rPA + 5 µg of rLF per rat).

## 3. Discussion

Because the lethality of anthrax is so high, it is desirable to develop a highly effective inhibitor against anthrax toxin. Many studies have shown that anthrax toxins must be transported into the cytoplasm by toxin receptors (CMG2 or TEM8), leading to toxicity [21,22]. Hence, the strategy of blocking the binding of PA to anthrax receptor is accepted as an effective method of inhibition. In this study, we focused on inhibiting the step in which PA binds to receptors on the cell surface. During this step, soluble CMG2 might compete with CMG2 for PA binding on the cell surface [8]. The results suggest HSA-CMG2 as a potential anthrax-specific therapeutic drug candidate.

To date, the development of anti-toxin therapies for anthrax has has mainly focused on monoclonal antibodies against the anthrax toxin components, including PA, LF, and EF [23]. Most antigen epitopes that have been targeted by these antibodies are similar, with some differences in their affinities and protective effects. In addition, receptor decoys also represent an important method for the development of anthrax-specific drugs. CMG2 was first identified as a receptor of *Bacillus anthracis* toxin by John A. T. Young in 2003, and its soluble form (sCMG2) was shown to protect CHO-K1 cells from attack by anthrax toxin [8] and prevent death in Fisher 344 rats [9]. Manchester and colleagues [17] speculated that anthrax toxin effected normally when the monoclonal antibody recognition sites were mutated by genetic engineering. The PA_L685A_ mutant abolished the protection effect of monoclonal antibody 14B7 but was still recognized by sCMG2, allowing sCMG2 to still inhibit the binding of PA to the membrane receptor and protect rats from death. A previous study showed that fusing the vWA domain of CMG2 with IgG-Fc could prolong the half-life of the receptor and protect mice from death [24]. The affinity of the CMG2-Fc mutant CMG2_E117Q_-Fc for rPA was increased four-fold using computer aided design, and the mutant was able to protect Fisher 344 rats from LT challenge for at least 24 h [25]. In contrast, different reports suggested that fusion of sCMG2 with Fc only extends the half-life of CMG2 and does not provide protection; this might be attributed to the formation of stable complexes of CMG2-Fc and PA in circulation followed by the slow release of PA, eventually leading to death [18]. Our finding that all the rats in the CMG2-Fc groups treated with LT died is consistent with the above study. In addition, Fc may be involved in ADCC (antibody-dependent cell-mediated cytotoxicity) and CDC (complement dependent cytotoxicity) *in vivo*, which do not affect the toxin inhibition activity of CMG2. Therefore, in this study, we constructed a new fusion protein HSA-CMG2 as an anthrax-specific therapeutic candidate drug with the hope of showing a good protective effect and prolonging the half-life *in vivo*. HSA, a 66.5-kDa non-glycosylation protein, makes up 60% of human serum and plays important roles in maintaining osmotic pressure and plasma volume, half-life of which is 14–20 days [26]. Hence, fusing with HSA may provide a way to increase the half-life of sCMG2, which can be expressed by a low-cost yeast expression system [27,28,29,30,31]. In the present study, when LT (10 μg rPA + 5 μg rLF per rat) was injected *in vivo*, HSA-CMG2 protected rats from the LT challenge for at least 14 days (Figure 4A). To determine whether HSA-CMG2 can protect rats under a higher dose of LT (20 μg rPA + 10 μg rLF per rat), a second *in vivo* LT neutralization assay was performed as above with increased doses of rPA and rLF. As expected, the HSA-CMG2 was able to prevent all of the rats from dying (Figure 4B). In this study, HSA-CMG2 was much more effective as a toxin inhibitor than CMG2-Fc. CMG2-Fc only prolonged the survival time, whereas HSA-CMG2 protected the rats for over 14 days. HSA-CMG2 also prolonged the half-life to 5 h *in vivo*, far greater than that of sCMG2 but lower than that of CMG2-Fc. It is possible that this intermediate half-life of HSA-CMG2 may prevent the slow release of toxin that occurs in the case of CMG2-Fc.

Although HSA and Fc share the same circulation mechanism, which is mediated by endosome and FcRn, the binding epitope is different and may exploit extinction kinetics. Interestingly, the pore formation and release of toxin into cell plasma was induced in the endosome by acidification. The early dissociation of the toxin receptor or pore formation without penetration into the membrane would eliminate toxicity and lead to the degradation of toxin proteins. The binding of toxin to CMG2-Fc may induce toxicity in a receptor-independent manner mediated by Fc receptor [32,33], although no evidence has been found for this idea. The structural pattern of HSA-CMG2 would exclude this possibility. To further examine the ability of HSA-CMG2 to neutralize LT, we performed additional experiments *in vivo*. When the LT (20 μg rPA + 10 μg rLF per rat) was injected before 5 min receptor decoy administration, both HSA-CMG2 and sCMG2 were able to prolong the survival time compared to the LT group (Figure 4C). When LT (10 μg rPA + 10 μg rLF per rat) was injected 5 min before HSA-CMG2 injection, only one rat in the HSA-CMG2 group died during the experiment (Figure 4D). These findings indicate that HSA-CMG2 can increase the survival times of the sick rats and even protects them from death. Since F344 rats are very sensitive to LT, we did not perform any tests in which LT was injected a longer time than 5 min before the injection of the receptor decoy. We also tested the injection of HSA-CMG2 (HSA-CMG2:rPA molar ratio = 10:1) 5 min or 24 h before LT administration. When LT was injected 5 min after receptor decoy administration, all rats were alive at the end of the trial (Figure 4E). This result was expected as the high injected dose of HSA-CMG2 or sCMG2 would have generated a sufficient receptor decoy concentration in the blood, neutralizing the toxin when LT was injected. In addition, when the receptor decoys were injected 24 h before LT administration, the survival time of the sCMG2 group equaled that of the LT group (*p* = 0.5988), while that of the HSA-CMG2 group was significantly longer than that of LT group (*p* = 0.0084) and that of the sCMG2 group (*p* = 0.0069; Figure 4F). This finding indicated that because the half-life of HSA-CMG2 is longer than that of sCMG2, the protection effect of HSA-CMG2 was superior to that of sCMG2 in this LT neutralization assay. Future studies are planned to evaluate the protection efficiency of HSA-CMG2 on animals infected with spores of *anthrax bacillus*.

In summary, we reported a receptor decoy, HSA-CMG2, that can bind PA with high affinity, neutralize LT, and protect J774A.1 cells and F344 rats from death related to anthrax toxin. We believe that the further development of HSA-CMG2 will allow it to be used in combination with antibiotics for the treatment of anthrax.

## 4. Experimental Section

### 4.1. Ethics Statement

Animal experiments were conducted in accordance with the recommendations of the Guide for the Care and Use of Laboratory Animals of the National Institutes of Health and approved by the Animal Ethics Committee of Beijing Institute of Biotechnology. In the half-life assay, the endpoint was the last blood sample collection time. The endpoint of *in vivo* toxin neutralization was when all experimental animals had died or were observed for 14 days. At the endpoint, living animals were euthanized via the injection of an overdose of pelltobarbitalum natricum.

### 4.2. Recombinant Anthrax Toxins

Recombinant protective antigen (rPA) and lethal factor (rLF) were expressed in *Escherichia coli* and purified as described previously [34,35].The purities of rPA (735 aa, 83-kDa) and rLF (776 aa, 90-kDa) were both estimated to be greater than 95% by SDS-PAGE.

### 4.3. Receptor Decoys Cloning

The DNA fragment of the vWA domain of CMG2 (sCMG2, aa 39–218 of CMG2, GenBank Accession Number AY233452) was amplified from cDNA maintained in our laboratory and cloned into PQE30 vector (QIAGEN, GmbH, Hilden, Germany) between *BamHI* and *HindIII* sites with a six-His tag at the 5′ end. Cys175 of sCMG2 was mutated to Ala to avoid the formation of interchain disulfide bonds. The primers 5′-*gctagtgtttatgctgttggtgtccttgattttgaac*-3′ and 5′-*ggacaccaacagcataaacact agccccaagtgac*-3′ were used to construct the Cys175-Ala175 mutation in the above plasmid, denoted as PQE30-sCMG2.

The plasmid CMG2-Fc was made by PCR amplification of the sCMG2 fragment (aa 1–219, 20-kDa) and the Fc fragment (human IgG1-Fc). The two fragments were linked by (G_4_S)_3_ and cloned into PCDNA3.1 (+) vector (Invitrogen, San Diego CA, USA) using *EcoRI* and *NotI* restriction enzymes.

The primers for HSA-CMG2 vector construction are listed in Table 3. First, the fragments of HSA (GenBank Accession Number AY542069, aa 25–609) and sCMG2 (aa 38–219) were amplified from cDNA maintained in our laboratory and PQE30-sCMG2, respectively, using H1F/H1R and CMG-F/CMG-R as the primers. Second, a part of the linker GGGGS×3 was ligated with or added onto the gene of sCMG2 by PCR using C-2F/CMG2-R as primers. The remaining part of the linker was ligated with the gene of HSA using H-1F/H-2R as primers. Third, the fused HSA-CMG2 gene was amplified by superimposed PCR using H-3F/C-3R as primers and the two linker-ligated PCR segments as templates. Finally, the HSA-CMG2 gene was ligated into the yeast vector pMEX9K (a patented expression vector developed by our lab) at the two enzyme sites *XhoI* and *NotI*.

All constructed plasmids were confirmed by sequencing (Taihe Bio. Co., Laiwu, China).

**Table 3:** Primers used for engineering the HSA-CMG2 plasmid.

Name	Sequences (5′-3′)
H-1F	*GCCACTCGAGAAAAGAGATGCACACAAGAGTGAGGTTGCTCATC*
H-1R	*CCGCCTGAACCGCCTCCACCTAAGCCTAAGGCAGCTTG*
CMG2-F	*GTGGCTCTGGCGGTGGCGGATCGTGCAGAAGAGCCTTTG*
CMG2-R	*GCTAGCCGAGCGGCCGCTTAACATGACTGAGCTAGTATAG*
H-2R	*CACCGCCAGAGCCACCTCCGCCTGAACCGCC*
C-2F	*TCAGGCGGAGGTGGCTCTGGCGGTG*
H-3F	*CATTCTCGAGAAAAGAGATGCACACAAGAGTG*
C-3R	*GAACGCGGCCGCTTAACATGACTG*

### 4.4. Receptor Decoys Expression and Purification

The sCMG2 was expressed in *E.coli strain* BL21 (DE3). After growth until OD_600_ = 0.6–0.8, the cell culture was induced with 0.4 mmol/L IPTG for 16 h at 16 °C. After ultra-sonication, the supernatant was collected, and sCMG2 was purified by chromatography using Ni-affinity column (GE Healthcare Bio-Sciences Corp., Piscataway, NJ, USA) and Source 30Q column (GE Healthcare Bio-Sciences Corp., Piscataway, NJ, USA). The purity of the sCMG2 was determined using 12% SDS-PAGE, Coomassie Brilliant Blue staining, and Western blot analysis.

The CMG2-Fc was expressed in Expi293F cells (Invitrogen, A14527, San Diego, CA, USA) using ExpiFectamine™ 293 Transfection Kits (Invitrogen, A14524). About 72 h after transfection, the cell supernatant was harvested and purified by affinity chromatography with a Protein G column (GE Healthcare) in accordance with the manufacturer’s purification system. The purity of the CMG2-Fc was determined using 12% SDS-PAGE, Coomassie Brilliant Blue staining.

The constructed HSA-CMG2 vectors were linearized and transformed to *pichia yeast* competent cells in an electroporation facility. After culturing at 30 °C for 48 to 72 h, positive clones were selected from MD plates. A single colony was inoculated into 25 mL BMGY in a 250 mL flask and cultured at 30 °C at 250 rpm to OD_600_ = 2–6. The overnight cultures were inoculated into 1 L BMGY in a 5 L flask and cultured at 30 °C at 250 rpm to OD_600_ = 2–6. The pellets were harvested by centrifugation at 2000× *g* for 5 min and re-suspended by 1 L BMMY. The suspensions were induced by 0.5% methanol at 28 °C for 72 h with the addition of methanol every 24 h to obtain a final concentration of 0.5%. The 1 L culture supernatant was then collected by centrifugation at 8000 g for 20 min, concentrated to 100 mL, and exchanged into buffer containing 20 mmol/L PB (pH = 7.4) by ultra-filtration. The processed supernatant was loaded onto a Blue Sepharose column (GE Healthcare) and eluted with 2 mol/L NaCl. The fractions were exchanged into 20 mmol/L PB by ultrafiltration, loaded onto a Q-HP column (GE Healthcare) in neutral pH buffer, and eluted with buffer containing 20 mmol/L PB + 1 mol/L NaCl. The protein was identified by 12% SDS-PAGE and Western blot analysis.

Finally, all proteins were concentrated and exchanged into PBS (pH 7.4), and their concentrations were determined using BCA protein assay (Pierce 23227, Rockford, IL, USA).

### 4.5. Surface Plasmon Resonance (SPR)

SPR experiments were performed on a BiacoreT200 (GE Healthcare) at 25 °C. The reagents used for coupling, binding, and regeneration were purchased from Biacore. The system was equilibrated with buffer HBS-P + containing 5 mmol/L Mg^2+^. rPA (30 μg) was coupled to the CM5 chip by amino coupling. For binding measurements, protein samples were injected into the channel at different concentrations at a flow rate of 20 μL/min. The fluid phase of the control channel was only HBS-P + 5 mmol/L Mg^2+^. All data were analyzed using BiacoreT200 evaluation software.

### 4.6. Half-Life Assay

Three male SD rats (200–220 g per rat) were treated with 200 μg HSA-CMG2 by tail vein injection. Blood samples were collected in heparinized Eppendorf tubes before injection and at different time points after injection. The plasma was isolated by centrifuging the blood samples at 4000× *g* at 4 °C for 15 min and stored at −20 °C before use. The method used to obtain plasma from injected CMG2-Fc rats was the same as in the HSA-CMG2 trials. The concentrations of HSA-CMG2 and CMG2-Fc in plasma were measured by double sandwich ELISA. Briefly, for HSA-CMG2, a 48-well plate was coated with 4 μg/mL anti-HSA (lifespan biosciences, LS-C51816) in PBS overnight at 4 °C; for CMG2-Fc, the plate was coated with 4 μg/mL anti-Fc (Prospec, ANT-173, East Brunswick, NJ, USA). The next day, the plates was washed four times with PBST and blocked with 2% BSA at 37 °C for 1 h. Then blood samples were added to plate wells washed with PBST and incubated at 37 °C for 1 h. After washing with PBST, polyclonal antibodies against CMG2 were added to the wells and incubated at 37 °C for 1 h. After washing with PBST, anti-rabbit HRP was added to wells and incubated for 1 h at 37 °C. Finally, TMB was added to determine the value of OD450/630 nm.

### 4.7. In Vitro Neutralization Assay (TNA)

The TNA experiment was performed as previously described [36]. Briefly, J774A.1 cells cultured in MEM containing 10% FBS (Fetal Bovine Serum) and 1% penicillin/streptomycin were seeded in 96-well plates to 70% confluence. Receptor decoys were diluted serially in complete medium containing rPA (50 ng/mL) and rLF (40 ng/mL). This mixture was applied to the cell plates and incubated for 4 h at 37 °C. The medium was replaced with 100 μL of fresh medium (2% FBS) containing 1 mg/mL MTT (3-(4,5-dimethyl-2-thiazolyl)-2,5-diphenyl-2-H-tetrazolium bromide) and then incubated for 1 h at 37 °C. After removing the medium and dissolving the produced blue pigment, cell viability was measured as the absorbance at 570 nm. Untreated cells and cells treated with only LT served as the controls.

### 4.8. In Vivo Protection against Intoxication

Forty male F344 rats (200–220 g each) were randomly divided into eight groups (five rats per group). Rats were treated with mixtures of 10 μg rPA, 5 μg rLF, and different doses of receptor decoys via intravenous injection in the tail (Table 2). HSA-IFN and LT were designed to be the controls. The survival time was recorded at different time points. To better reflect the maximum PA concentrations after infection with *B. anthracis*, a second *in vivo* LT neutralization assay was performed as above with increased doses of rPA and rLF (20 and 10 μg per rat, respectively). Further, the rats were also treated with LT 5 min before receptor decoys injection. For this experiment, the male F344 rats (200–220 g) were injected intravenously with PBS, HSA-CMG2 (the receptor decoy: rPA molar ratio, 10:1) or sCMG2 (the receptor decoy: rPA molar ratio, 10:1) after receiving an intravenous injection of LT (20 μg rPA + 10 μg rLF or 10 μg rPA + 10μg rLF per rat), respectively. Additionally, HSA-CMG2 and sCMG2 were injected to test their abilities to neutralize LT. Male F344 rats (200–220 g) were inoculated with PBS, HSA-CMG2 (receptor decoy:rPA molar ratio = 10:1), or sCMG2 (receptor decoy:rPA molar ratio = 10:1) followed by LT (20 μg rPA + 10 μg rLF per rat) administration after 5 min or 24 h.

### 4.9. Statistical Analysis

All SPR data were analyzed using BiacoreT200 evaluation software. The results represent three independent determinations and are shown as mean ± SD. The half-life data were calculated using one-phase exponential decay with transform *y* = log[*y*]/log[2] followed by the linear regression of the transform data. The half-life was determined as the negative reciprocal of the slope of the line. The IC_50_ values for the *in vitro* toxin neutral assays were determined using the unpaired Student’s *t*-test. Survival curves were analyzed by log-rank test (Mantel-Cox) using GraphPad Prism software. Statistical analysis of toxin neutralization *in vivo* was performed using unpaired Student’s *t*-test.
